# Supplementary material for: CopulaNet: Learning residue co-evolution directly from multiple sequence alignment for protein structure prediction
Source: Nat Commun. 2021 May 5;12:2535. doi: 10.1038/s41467-021-22869-8 (PMC8100175; doi:10.1038/s41467-021-22869-8)
Supplement: Supplementary file 1 — Supplementary Information [file 41467_2021_22869_MOESM1_ESM.pdf]

CopulaNet: Learning residue co-evolution directly from multiple sequence alignment for protein structure prediction

Fusong Ju[1,2], Jianwei Zhu[3,*], Bin Shao[3], Lupeng Kong[1,2], Tie-Yan Liu[3], Wei-Mou Zheng[4,2], and Dongbo Bu[1,2,*]

[1] Key Lab of Intelligent Information Processing, State Key Lab of Computer Architecture, Big-data Academy, Institute of Computing Technology, Chinese Academy of Sciences, Beijing 100190, China. [2] University of Chinese Academy of Sciences, Beijing 100049, China. [3] Microsoft Research Asia, Beijing 100080, China. [4] Institute of Theoretical Physics, Chinese Academy of Sciences, Beijing 100190, China.

Supplementary Material

Supplementary Methods

Supplementary Table 1: Precision of the inter-residue contact predictions by RaptorX, A7D, trRosetta, and ProFOLD over CASP13 targets

Supplementary Table 2: Comparison of ProFOLD and baseline models in terms of precision of contact predictions

Supplementary Table 3: Quality of the predicted tertiary structures for CASP13 targets. Here, we list TMscore of top1/top5 predicted structure

Supplementary Table 4: The performance of ProFOLD under various settings of hyperparameters

Supplementary Figure 1: Predict tertiary structure for CASP13 FM target protein T0992-D1 using ProFOLD

Supplementary Figure 2: Outer product of the embedding features and average pooling in the co-evolution aggregator module

Supplementary Figure 3: Quality of the predicted structure for the CASP13 FM target proteins

Supplementary Figure 4: Head-to-head comparison of trRosetta and ProFOLD on the CASP13 FM target proteins

Supplementary Figure 5: Precision of the inter-residue contact predictions by ProFOLD and the variant *ProFOLD w/o OP*

Supplementary Figure 6: Architecture of the residual blocks used by *MSA encoder* and *distance estimator*

Supplementary Methods

Hyperparameters of the neural networks used by CopulaNet

- 1D ResNet: 8 one-dimensional blocks with 64 channels.
- 2D ResNet: 18 groups of 4 two-dimensional blocks with 96 channels, cycling through dilations 1, 2, 4, 8.
- Optimizer: Adam with learning rate $1e^{-3}$.
- Batch: mini-batch of 3 crops on each of 4 GPU workers.
- Nonlinearity: ELU.
- Loss: cross-entropy.
- Training time: about 15 hours for 60,000 steps.

Evaluation criteria of the predicted structures and inter-residue distances

We evaluated the quality of the predicted structures by aligning them with the native structure using DeepAlign [1]. For the CASP13 targets, DeepAlign reports TMscore [2] of the predicted structures, which are close to the TMscore published by CASP13 organizer. For each target protein, we predicted multiple tertiary structures (called `models`), and, following the CASP convention, evaluated the prediction approaches in terms of both top 1 and top 5 models.

For the sake of fair comparison, we evaluated the estimated inter-residue distances through transforming into inter-residue contacts. Specifically, for two residues, we summed up the predicted probability mass of distance below 8Å, and used the sum as the predicted probability for the two residue being in contact. We calculated the precision of the most probable $L/5$, $L/2$ and L predicted contacts, where L denots the length of protein sequence [3]. In the study, we focused on the long-range contacts (between two residues with sequence separation over 24 residues) as they are harder to predict and more important for constructing structure.

The calculation process of outer product

Consider a target protein with L residues. The alignment of a homologous protein with target protein might have insertions, deletions, and mutations. As our objective is to describe residue co-mutations, we discarded all insertions by following the conventions used by Ref. [4]. Thus, a homologous protein can be represented as a L-long sequence composing of residues and a special character '-' representing deletion, and we can assume that each homologous protein is a random variable $R = r_1 r_2 \cdots r_L$ sampled from the joint distribution $P(r_1, r_2, ..., r_L)$.

As mentioned in the main text, from the k-th homologous protein, the MSA encoder extracts the mutations of the i-th target residue r_i and embeds these mutations into a vector $X_k(i)$ using a CNN. Previous studies have already suggested that, although in principle CNN has the ability to describe long range relationship, it focuses on neighboring residues rather than distant pairs [5]. Thus, to be more precise, we rewrite $X_k(i)$ as $X_k(i, N_i)$, where N_i represents the neighboring residues of r_i that have considerable effects on its mutation. Accordingly, we rewrite the aggregated embedding feature $f(i)$ as $f(i, N_i)$, and rewrite the aggregated outer product $g(i, j)$ as $g(i, N_i, j, N_j)$, which can be calculated as follows:

$$g(i, N_i, j, N_j) = \frac{1}{M_{eff}} \sum_{k=1}^{K} w_k [X_k(i, N_i) \otimes X_k(j, N_j)]. \quad (1)$$

Here, the symbol "$\otimes$" represents outer product operation. Outer product could effectively capture the correlation between two variables. To understand how outer product works, we showed in Supplementary Figure 2 the outer product of two one-hot feature vectors as an illustrative example. In this case, the 3rd entry of the one-hot vector $f(i, N_i)$ is 1, and the 1st entry of the one-hot vector $f(j, N_j)$ is 1. Thus, the outer product of them has 1 at the (3, 1)-th entry, which clearly reveals the correlation between these vectors. In practice, instead of the simple one-hot feature vectors, the neural network uses real-value feature vectors.

Through averaging over all homologous proteins, the aggregated outer product $g(i, N_i, j, N_j)$ is expected to approximate the joint distribution $P(x_i, N_i, x_j, N_j)$. Similarly, the neural network could also approximate the joint distribution $P(N_i, N_j)$. Thus, the outer product is expected to provide information of the conditional joint-residue distribution $P(x_i, x_j | N_i, N_j)$.

Implementation of baseline models

We implemented three baseline models, including "CCMpred+2DResNet" (denoted as *baseline-CCM*), "Covariance matrix+2DResNet" (denoted as *baseline-Cov*), and "comprehensive features+2DResNet" (denoted as *baseline-CF*) by following RaptorX and AlphaFold. The training set, input features and architecture of neural network are described in details as below.

1. *Dataset:* We use exactly the same protein list as AlphaFold and ProFOLD to train these baseline models. Briefly speaking, the benchmark dataset was constructed through utilizing 35% sequence similarity cluster representatives of CATH (as of Mar. 16, 2018) [6]. It contains a total of 31,247 non-redundant domains, which was further partitioned into training and validation sets (containing 29,247 and 1,820 proteins, respectively). During the partitioning process, all domains from the same homologous superfamily were allocated to the same partition, thus avoiding potential overlap between partitions.

2. *Input features:* For a target protein with L residues, the input features of baseline-CCM consist of CCMpred output (a full $L \times L \times 21 \times 21$ matrix) together with sequence profile features. Unlike RaptorX, baseline-CCM does not use extra features such as predicted secondary structure, accessible surface area and mutual information.

   The input features of baseline-Cov consist of covariance matrix calculated from target MSA (a full $L \times L \times 21 \times 21$ matrix) together with sequence profile features. Similarly to baseline-CCM, baseline-Cov also does not use extra features such as predicted secondary structure, accessible surface area and mutual information.

   Unlike baseline-CCM and baselin-Cov, baseline-CF uses comprehensive features, including amino acids types, sequence profile, predicted secondary structure, mutual information, covariance matrix (a full $L \times L \times 21 \times 21$ matrix) and CCMpred output (also a full $L \times L \times 21 \times 21$ matrix).

3. *Architecture of neural network:* The architecture of 2D ResNet in these baseline models is similar to that of RaptorX and AlphaFold. The only difference lies at the number of convolutional layers: our baseline models use 144 convolutional layers whereas AlphaFold uses about 600 layers and RaptorX uses about 60 layers.

Comparison with the updated RaptorX

We also compared ProFOLD with the newly-updated RaptorX (denoted as RaptorX2020 in this manuscript; see Ref. [7]). Specifically, we downloaded the released model of RaptorX2020 (through https://github.com/j3xugit/RaptorX-3DModeling) and evaluated it using identical MSA to ProFOLD. The results are summarized in Supplementary Table 1.

As shown in this table, on the 31 CASP13 FM targets, ProFOLD achieved higher precision for long-range contact predictions than RaptorX2020 (0.567 vs. 0.537, 0.713 vs. 0.689, and 0.840 vs. 0.801 for the most probable L, $L/2$ and $L/5$ contacts, respectively). On the 12 FM/TBM and 61 TBM targets, the advantage of ProFOLD is more significant.

Supplementary Tables

Table 1 | Precision of the inter-residue contact predictions by RaptorX, A7D, trRosetta, RaptorX2020 and ProFOLD over CASP13 targets. Here, the precision for the most probable L, $L/2$ and $L/5$ long-range contacts over CASP13 targets are shown. RaptorX2020 denotes the updated RaptorX. Following the CASP13 criteria, T0953s1 is excluded as it has no long-range contacts

	FM (31 domains)			FM/TBM (12 domains)			TBM (61 domains)		
Method	L	$L/2$	$L/5$	L	$L/2$	$L/5$	L	$L/2$	$L/5$
RaptorX	0.439	0.562	0.686	0.530	0.645	0.807	0.652	0.798	0.899
A7D	0.470	0.596	0.712	0.584	0.731	0.866	0.694	0.826	0.901
trRosetta	0.489	0.627	0.750	0.576	0.728	0.867	0.649	0.792	0.917
RaptorX2020	0.537	0.689	0.801	0.583	0.758	0.902	0.634	0.773	0.878
ProFOLD	**0.567**	**0.713**	**0.840**	**0.646**	**0.791**	**0.913**	**0.717**	**0.860**	**0.948**

Table 2 | Comparison of ProFOLD and baseline models in terms of precision of contact predictions. Here, the precision for the most probable L, $L/2$ and $L/5$ long-range contacts over CASP13 targets and validation set are shown. The “shallow” 2DResNet has a total of 36 residual blocks whereas 2DResNet in ProFOLD has a total of 72 residual blocks. The baseline model baseline-CCM trains our 2D ResNet using CCMpred output (a $L \times L \times 21 \times 21$ matrix) together with sequence profile as input features, whereas baseline-Cov trains our 2D ResNet using covariance matrix (also a $L \times L \times 21 \times 21$ matrix) together with sequence profile as input features. The baseline model baseline-CF uses comprehensive features, including amino acid types, sequence profile, predicted secondary structure, mutual information, covariance matrix and CCMpred output. Following the CASP13 criteria, T0953s1 is excluded as it has no long-range contacts

	CASP13 FM (31 domains)			Validation set (1820 domains)		
Method	L	$L/2$	$L/5$	L	$L/2$	$L/5$
baseline-CCM	0.466	0.603	0.738	0.582	0.764	0.871
baseline-Cov	0.449	0.591	0.713	0.556	0.735	0.852
baseline-CF	0.481	0.621	0.749	0.595	0.778	0.879
ProFOLD	0.567	0.713	0.840	0.641	0.831	0.918
Shallow baseline-CCM	0.445	0.582	0.702	0.569	0.754	0.857
Shallow baseline-Cov	0.424	0.570	0.687	0.541	0.722	0.838
Shallow baseline-CF	0.458	0.595	0.712	0.572	0.763	0.869
Shallow ProFOLD	0.544	0.689	0.808	0.632	0.819	0.910

Table 3 | Quality of the predicted tertiary structures for CASP13 targets. Here, we list TMscore of top1/top5 predicted structure. For trRosetta and ProFOLD, we show their performance with two types of MSAs as inputs, namely, “target MSA" represents the MSAs constructed through searching the whole-target sequence against sequence database, whereas “domain MSA" represents the MSAs constructed through searching domain sequences against sequence database

Method	All (104)	TBM (61)	FM/TBM(12)	FM (31)
A7D	0.699/0.733	0.761/0.786	0.691/0.739	0.580/0.626
Zhang (Human)	0.692/0.719	**0.801**/0.816	0.605/0.665	0.509/0.549
MULTICOM (Human)	0.688/0.722	0.794/**0.817**	0.645/0.675	0.495/0.551
QUARK	0.672/0.699	0.786/0.808	0.589/0.648	0.479/0.503
Zhang-Server	0.671/0.699	0.787/0.807	0.593/0.627	0.475/0.514
RaptorX-DeepModeller	0.653/0.674	0.774/0.786	0.561/0.592	0.451/0.486
trRosetta (target MSA)	0.620/0.643	0.651/0.678	0.597/0.607	0.567/0.587
ProFOLD (target MSA)	0.719/0.727	0.747/0.755	0.696/0.704	**0.671/0.680**
trRosetta (domain MSA)	0.668/0.677	0.719/0.727	0.622/0.625	0.584/0.599
ProFOLD (domain MSA)	**0.743/0.751**	0.784/0.793	**0.740/0.744**	0.662/0.673

Table 4 | The performance of ProFOLD under various settings of hyper-parameters. Here, the precision for the most probable L, $L/2$ and $L/5$ long-range contacts over CASP13 FM targets and validation set are shown. Following the CASP13 criteria, T0953s1 is excluded as it has no long-range contacts

Method	#Residual blocks	#Channels	#Parameters	CASP13 FM (31 domains)			Validation set (1820 domains)		
				L	$L/2$	$L/5$	L	$L/2$	$L/5$
Shallow ProFOLD	36	96	6.46 M	0.544	0.689	0.808	0.632	0.819	0.910
Shallow but wide ProFOLD	36	128	11.19 M	0.548	0.688	0.811	0.635	0.821	0.909
ProFOLD	72	96	12.44 M	0.567	0.713	0.840	0.641	0.831	0.918
Deeper ProFOLD	96	96	16.43 M	0.570	0.714	0.839	0.643	0.833	0.922

Supplementary Figures

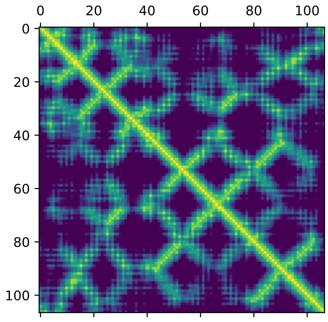

(a) Native distance

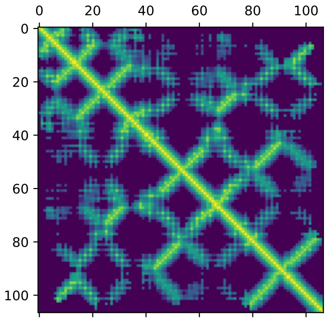

(b) Predicted distance

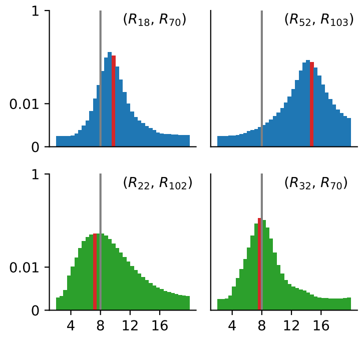

(c) Distribution

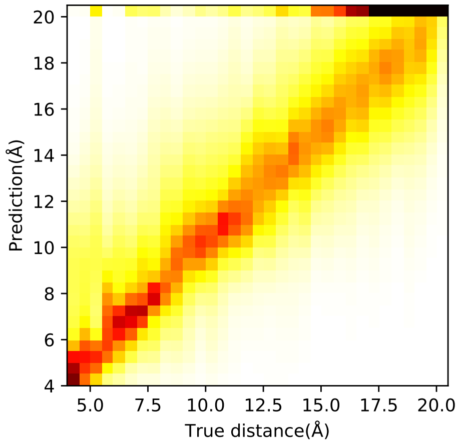

(d) Predicted distribution

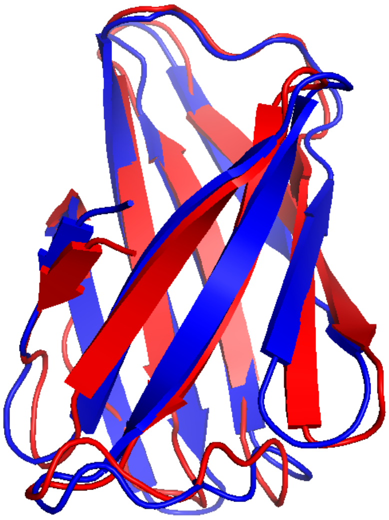

(e) Native structure

Figure 1 | Predict tertiary structure for CASP FM target protein T0992-D1 using ProFOLD. (a) The native inter-residue distances. (b) The predicted inter-residue distances using CopulaNe, which is very close to the native distances. (c) The predicted inter-residue distance distribution for 4 residue pairs. Here, the bin covering the ground-truth distance is highlighted in red. True-contact distributions are plotted in green, otherwise in blue. The gray line represents the contact cutoff 8Å. (d) The comparison of the predicted distance distributions and the ground-truth distances. The concentration on the diagonal line suggests that the predicted distances are very close to the ground-truth distances. (e) The predicted tertiary structure (in blue) and the native structure (in red). The TMscore between them is 0.84, indicating that the predicted structure has high quality

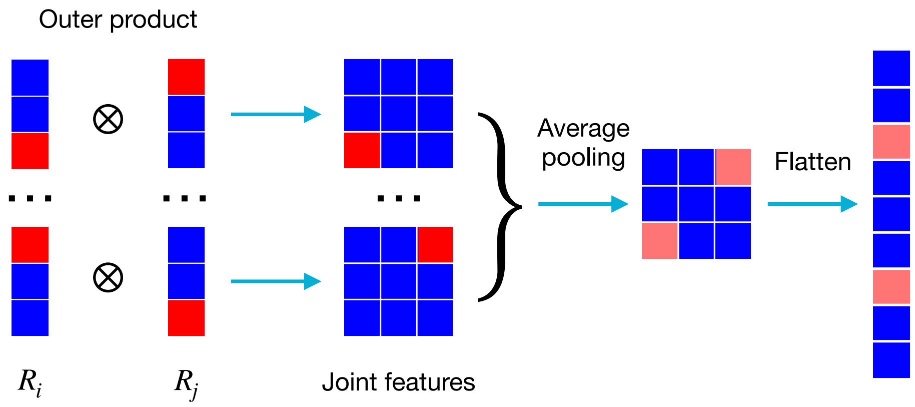

Figure 2 | Outer product of the embedding features and average pooling in the co-evolution aggregator module. To understand how outer product works, we showed here the outer product of two one-hot feature vectors as an illustrative example. For the 1st homologue protein, the 3rd entry of the one-hot vector for R_i is 1, and the 1st entry of the one-hot vector for R_j is 1. Thus, the outer product of them has 1 at the $(3, 1)$-th entry, which clearly reveal the correlation between these vectors. Next, we use average pooling to combine the outer products calculated for all homologue proteins. In practice, instead of the simple one-hot feature vectors, more informative real-value feature vectors are used.

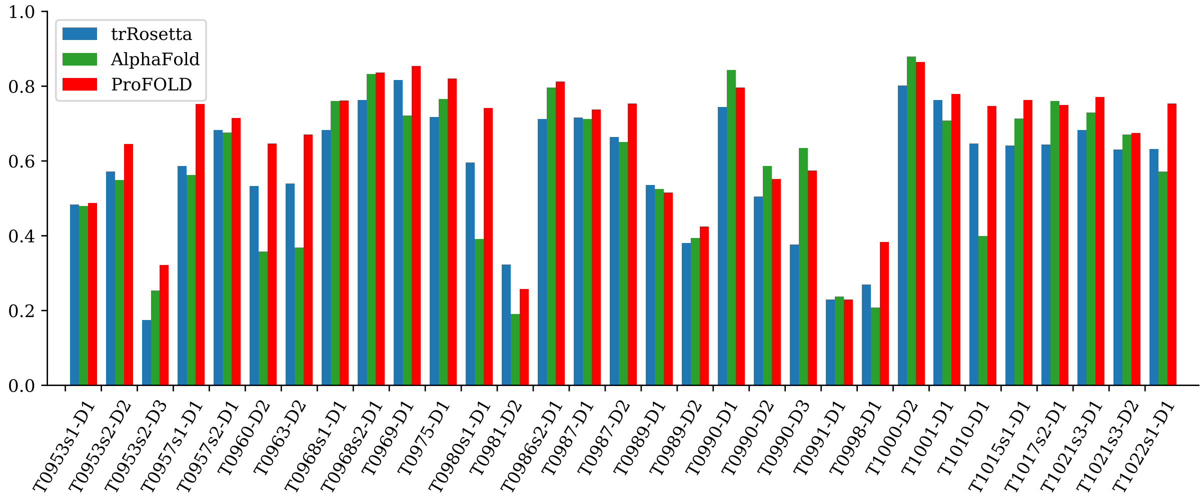

Figure 3 | Quality of the predicted structure for the CASP13 FM targets. Here we show TMscore of the top 1 model predicted by trRosetta, AlphaFold and ProFOLD

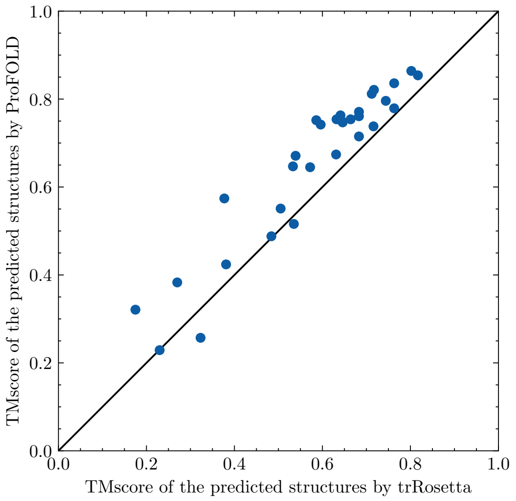

Figure 4 | Head-to-head comparison of trRosetta and ProFOLD on the CASP13 FM targets. Here we use TMscore of the top 1 model predicted by the two approaches. For 27 out of the 31 FM targets, ProFOLD outperformed trRosetta

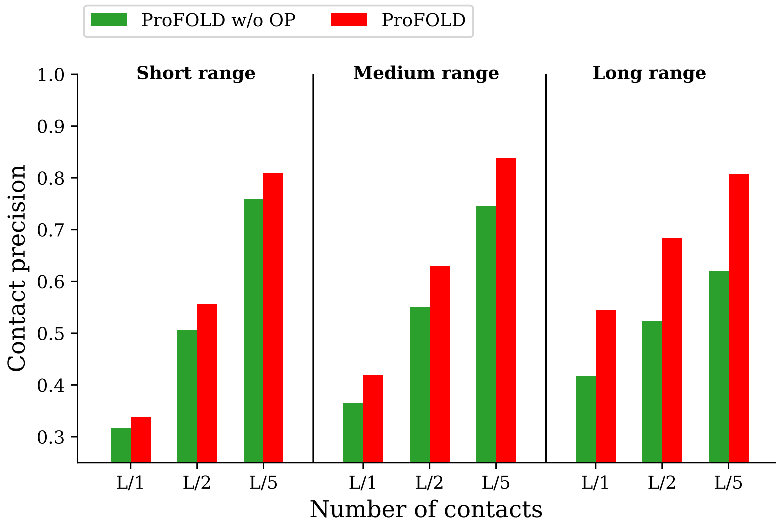

Figure 5 | Precision of the inter-residue contact predictions by ProFOLD and the variant *ProFOLD w/o OP* over 31 CASP13 FM targets. Here, the variant *ProFOLD w/o OP* was built through disabling the outer product operation of ProFOLD. For the short-range residue contacts (between two residues with sequence distance from 6 to 11 residues), *ProFOLD w/o OP* showed roughly the same prediction precision as ProFOLD. This is reasonable as the convolution modules in MSA encoder has already effectively modeled the short range relationship. In contrast, for the long-range residue contacts, the prediction accuracy of *ProFOLD w/o OP* decreased sharply to be significantly lower than ProFOLD. This result clearly demonstrated the importance of the outer product operation in modeling the long-range residue contacts, which cannot be achieved using the convolutional network alone.

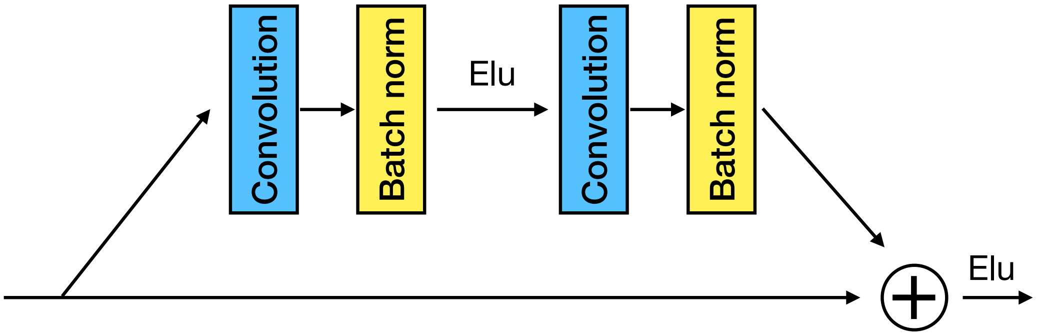

Figure 6 | Architecture of the residual blocks used by *MSA encoder* and *distance estimator*. The residual network in *MSA encoder* has 8 residual blocks, and each residual block consists of two batch-norm layers, two 1D convolution layers with 64 filters (with kernel size of 3) and exponential linear unit (ELU) nonlinearities. *Distance estimator* has 72 residual blocks with roughly same components, but use 2D 3×3 dilated convolution layers with 96 filters.

References

[1] S. Wang et al. Protein structure alignment beyond spatial proximity. *Scientific Reports* **3**, p. 1448 (2013).

[2] Y. Zhang and J. Skolnick. Scoring function for automated assessment of protein structure template quality. *Proteins: Structure, Function, and Bioinformatics* **57** no. 4, pp. 702–710 (2004).

[3] S. Wang et al. Accurate de novo prediction of protein contact map by ultra-deep learning model. *PLoS Computational Biology* **13** no. 1, p. e1005 324 (2017).

[4] M. Weigt et al. Identification of direct residue contacts in protein–protein interaction by message passing. *Proceedings of the National Academy of Sciences* **106** no. 1, pp. 67–72 (2009).

[5] W. Luo et al. Understanding the effective receptive field in deep convolutional neural networks. In D. D. Lee et al. (eds.), *Advances in Neural Information Processing Systems 29*, pp. 4898–4906 (Curran Associates, Inc., 2016).

[6] N. L. Dawson et al. CATH: an expanded resource to predict protein function through structure and sequence. *Nucleic Acids Research* **45** no. D1, pp. D289–D295 (2017).

[7] J. Xu, M. Mcpartlon and J. Li. Improved protein structure prediction by deep learning irrespective of co-evolution information. *bioRxiv* E-print, URL `https://www.biorxiv.org/content/early/2020/10/12/2020.10.12.336859`.
