# Supplementary material for: CopulaNet: Learning residue co-evolution directly from multiple sequence alignment for protein structure prediction
Source: Nat Commun. 2021 May 5;12:2535. doi: 10.1038/s41467-021-22869-8 (PMC8100175; doi:10.1038/s41467-021-22869-8)
Supplement: Supplementary file 3 — Reporting Summary [file 41467_2021_22869_MOESM3_ESM.pdf]

Corresponding author(s): Dongbo Bu
Last updated by author(s): Mar 22, 2021

Reporting Summary

Nature Research wishes to improve the reproducibility of the work that we publish. This form provides structure for consistency and transparency in reporting. For further information on Nature Research policies, see our Editorial Policies and the Editorial Policy Checklist.

Statistics

For all statistical analyses, confirm that the following items are present in the figure legend, table legend, main text, or Methods section.

n/a	Confirmed	
☐	☒	The exact sample size (*n*) for each experimental group/condition, given as a discrete number and unit of measurement
☐	☒	A statement on whether measurements were taken from distinct samples or whether the same sample was measured repeatedly
☐	☒	The statistical test(s) used AND whether they are one- or two-sided<br>*Only common tests should be described solely by name; describe more complex techniques in the Methods section.*
☐	☒	A description of all covariates tested
☐	☒	A description of any assumptions or corrections, such as tests of normality and adjustment for multiple comparisons
☒	☐	A full description of the statistical parameters including central tendency (e.g. means) or other basic estimates (e.g. regression coefficient) AND variation (e.g. standard deviation) or associated estimates of uncertainty (e.g. confidence intervals)
☒	☐	For null hypothesis testing, the test statistic (e.g. *F*, *t*, *r*) with confidence intervals, effect sizes, degrees of freedom and *P* value noted<br>*Give P values as exact values whenever suitable.*
☒	☐	For Bayesian analysis, information on the choice of priors and Markov chain Monte Carlo settings
☒	☐	For hierarchical and complex designs, identification of the appropriate level for tests and full reporting of outcomes
☐	☒	Estimates of effect sizes (e.g. Cohen's *d*, Pearson's *r*), indicating how they were calculated

Our web collection on statistics for biologists contains articles on many of the points above.

Software and code

Policy information about availability of computer code

Data collection	We used the following tools to collect and pre-process the data: PDB 2018-03, CATH 2018-03, DeepMSA 2020-02, PyRosetta v2020.19. Please see the methods section for more details.
Data analysis	The network was trained using PyTorch v1.4. Both network and full protein structure prediction pipeline are available via https://github.com/fusong-ju/ProFOLD. We used the following tools to analysis: PyMol v2.3.0, DeepAlign v1.135.

For manuscripts utilizing custom algorithms or software that are central to the research but not yet described in published literature, software must be made available to editors and reviewers. We strongly encourage code deposition in a community repository (e.g. GitHub). See the Nature Research guidelines for submitting code & software for further information.

Data

Policy information about availability of data

All manuscripts must include a data availability statement. This statement should provide the following information, where applicable:

- Accession codes, unique identifiers, or web links for publicly available datasets
- A list of figures that have associated raw data
- A description of any restrictions on data availability

Our training, validation and test data splits are available via http://protein.ict.ac.cn/ProFOLD. The following versions of public datasets were used in this study: PDB 2018-03; CATH 2018-03; Uniclust30 2017-10; UniRef90 2018-03; and Metaclust 2018-01.

Field-specific reporting

Please select the one below that is the best fit for your research. If you are not sure, read the appropriate sections before making your selection.

☒ Life sciences ☐ Behavioural & social sciences ☐ Ecological, evolutionary & environmental sciences

For a reference copy of the document with all sections, see nature.com/documents/nr-reporting-summary-flat.pdf

Life sciences study design

All studies must disclose on these points even when the disclosure is negative.

Sample size	Two test sets are used in this study.<br>The principal set consisted of 104 CASP13 domains as segmented by the CASP13 assessors.<br>The other set of 1820 domains was extracted from CATH. During the partitioning process, all domains from the same homologous superfamily were allocated to the same partition, thus avoiding potential overlap between train and test sets.
Data exclusions	Some CASP13 targets were excluded by the CASP13 assessors because of publications.<br>Server-only targets were excluded for fair comparison.
Replication	All source codes and benchmark data are available, so results are reproducible.<br>Multiple networks were trained independently and were found to give consistent results over both validation and test sets.
Randomization	Superfamilies were randomly assigned to CATH train or CATH test set.<br>CASP13 assessors determined the CASP13 test set manually and assigned the 104 domains to FM, TBM, or FM/TBM category.
Blinding	The investigators were not blind to the CASP13 test set. But all data used in this study, include PDB and sequences databases, are released before CASP13 and thus there is no overlap between training and CASP test set.

Reporting for specific materials, systems and methods

We require information from authors about some types of materials, experimental systems and methods used in many studies. Here, indicate whether each material, system or method listed is relevant to your study. If you are not sure if a list item applies to your research, read the appropriate section before selecting a response.

Materials & experimental systems

n/a	Involved in the study	
☒	☐	Antibodies
☒	☐	Eukaryotic cell lines
☒	☐	Palaeontology and archaeology
☒	☐	Animals and other organisms
☒	☐	Human research participants
☒	☐	Clinical data
☒	☐	Dual use research of concern

Methods

n/a	Involved in the study	
☒	☐	ChIP-seq
☒	☐	Flow cytometry
☒	☐	MRI-based neuroimaging
